# Combined use of probucol and cilostazol with atorvastatin attenuates atherosclerosis in moderately hypercholesterolemic rabbits

**DOI:** 10.1186/s12944-015-0083-5

**Published:** 2015-07-29

**Authors:** Yanli Wang, Liang Bai, Yan Lin, Yulong Chen, Hua Guan, Ninghong Zhu, Yafeng Li, Shoucui Gao, Lijing Sun, Sihai Zhao, Jianglin Fan, Enqi Liu

**Affiliations:** Research Institute of Atherosclerotic Disease, Xi’an Jiaotong University Cardiovascular Research Center, Xi’an, Shaanxi 710061, China; Department of Pathology, Xi’an Medical University, Xi’an, Shaanxi 710021 China; Laboratory Animal Center, Xi’an Jiaotong University School of Medicine, Xi’an, Shaanxi 710061 China; Shaanxi Key Laboratory of Ischemic Cardiovascular Disease, Institute of Basic and Translational Medicine, Xi’an Medical University, Xi’an, Shaanxi 710021 China; Department of Molecular Pathology, Interdisciplinary Graduate School of Medicine and Engineering, University of Yamanashi, Yamanashi, 409-3898 Japan

**Keywords:** Atorvastatin, Probucol, Cilostazol, Atherosclerosis, Rabbits

## Abstract

**Background:**

Atherosclerotic cardiovascular disease is one of the major diseases that seriously impacts human health. Combined drug therapy may be efficacious in delaying the occurrence of cardiovascular events.

**Aim:**

The current study was designed to investigate whether combined use of probucol (an anti-oxidant agent) with cilostazol (a platelet aggregation inhibitor) would increase the inhibitory effect of statins (a lipid-lowering agent) on atherosclerosis in moderately hypercholesterolemic rabbits.

**Methods and Results:**

Thirty Japanese white rabbits were fed with a high cholesterol diet for 12 weeks, which was supplemented with either 0.005 % atorvastatin alone or 0.005 % atorvastatin plus 0.3 % probucol and 0.3 % cilostazol. Except for high-density lipoprotein cholesterol, no difference was found in plasma lipids among vehicle, statin, and the combined treatment group. However, atherosclerotic lesions were significantly reduced by statin treatment compared with vehicle. Moreover, we found that the anti-atherogenic effect of statin was further enhanced by the combined treatment, which was due to increased anti-inflammatory and anti-oxidant properties.

**Conclusions:**

These data demonstrated that combined drug treatment exhibits potent athero-protective effects via pleiotropic functions, such as anti-inflammatory and anti-oxidative stress, which is independent of the lipid-lowering effect.

## Introduction

Atherosclerotic cardiovascular disease (ASCVD) is the foremost cause of disability and mortality in both developed and developing countries [1–3]. Atherosclerosis is a multifactorial disease and progresses slowly throughout the human life; therefore treatment of atherosclerosis requires many therapeutic strategies. Statins, hydroxylmethyl glutaryl coenzyme A reductase inhibitors, are widely used for treating hyperlipidemia [4–7]. Although statins are the first choice for treatment of atherosclerosis [8–11], there are still many patients who are not responsive to statins. Actually, statins intolerance is frequently encountered in clinical practice [12, 13]. Therefore, it may be practical to consider the combined use of statins with other drugs for those unresponsive patients. Previous studies have shown that atorvastatin combined with probucol exhibited a stronger anti-atherogenic effect than single drug treatment [14]. Probucol, a diphenolic compound with anti-inflammatory and anti-oxidant properties, can reduce atherosclerosis and restenosis in coronary arteries [15, 16]. Cilostazol, an inhibitor of type 3 phosphodiesterase, is widely used for treating thrombotic vascular disease and exerts antiplatelet activity via suppression of cyclic adenosine monophosphate degradation [17, 18]. Our previous studies found that the combined use of probucol with cilostazol has a greater anti-atherogenic effect than single probucol treatment [19]. Rabbits fed with a cholesterol diet are readily to develop atherosclerotic lesions, which mimics the lesions observed in ASCVD patients [20]. However, whether combination of statins with probucol and cilostazol has an add-on effect on atherosclerosis in moderately hypercholesterolemic rabbits is still unknown.

In the current study, a rabbit model with moderately hypercholesterolemia was established by feeding a cholesterol diet. The effect of statins combined with probucol and cilostazol on atherosclerosis was investigated. We found that combined drug treatment significantly attenuated atherosclerosis through inhibiting anti-inflammatory and antioxidant properties independent of the lipid-lowering function.

## Materials and methods

### Animals and diets

Thirty Japanese white rabbits (male, 4-mon) were supplied by Vital River Laboratories, Beijing, China. The rabbits were randomly divided into three groups: vehicle group (*n* = 10); atorvastatin group (0.005 % atorvastatin, *n* = 10); and APC group (0.005 % atorvastatin + 0.3 % probucol + 0.3 % cilostazol, *n* = 10). All rabbits were fed a cholesterol diet containing 0.3 % cholesterol and 3 % soybean oil for 12 weeks.

The probucol and cilostazol were provided by Otsuka Pharmaceutical Co., Ltd. Tokushima, Japan. Atorvastatin calcium was purchased from Sequoia Research Products Ltd, Pangbourne, UK. The drugs were mixed with cholesterol diets and prepared by Ke’ao Xieli Diet Co., Ltd., Beijing, China. The drug concentrations in diet were measured using gas chromatography or high-performance liquid chromatographic methods as previously described [21, 22]. All rabbits were given a restricted diet (100 g/rabbit per day) and free access to water. The animal experiments were approved by the Laboratory Animal Administration Committee of Xi’an Jiaotong University and carried out according to the Guidelines for Animal Experimentation of Xi’an Jiaotong University and the Guide for the Care and Use of Laboratory Animals published by the US National Institutes of Health (NIH Publication NO. 85–23, revised 2011).

### Determination of plasma lipid levels and other biochemical parameters

Blood samples were collected from the ear artery using an EDTA anticoagulant tube after 16 h fasting. Plasma was obtained after centrifuging at 3000 rpm for 20 min. The plasma triglycerides (TG), total cholesterol (TC), high-density lipoprotein cholesterol (HDL-C) and low-density lipoprotein cholesterol (LDL-C) were determined by commercial assay kits (Biosino Bio-technology and Science Inc., Beijing, China). Plasma TC and TG levels were measured biweekly, while plasma HDL-C and LDL-C levels were measured every 4 weeks. In order to compare whole plasma lipid levels in 12 weeks among three groups, the incremental area under the curve (AUC) was calculated according to the trapezium rule [23].

The C-reactive protein (CRP) levels were quantified using an ELISA kit (Immunology Consultants Laboratory, Inc., Newberg, OR, USA). The plasma levels of superoxide dismutase (SOD) and malondialdehyde (MDA) were measured by xanthine oxidase assay and thiobarbituric acid assay kits (Nanjing Jiancheng Bioengineering Institute, Nanjing, China), respectively, and oxidized LDL (ox-LDL) was measured by an ELISA kit (R&D Systems, Minneapolis, MN,USA).

### Quantification of gross atherosclerotic lesions

At the end of the experiment, all rabbits were euthanized by intravenous injection of an overdose of sodium pentobarbital. Rabbit aortas were subsequently collected for analysis of the aortic lesions. Aortic *en face* atherosclerosis was evaluated after the aortic trees were stained with Sudan IV as previously described [24]. Sudanophilic area was quantified using image analysis software (WinROOF Ver.6.5, Mitani Co., Ltd., Fukui, Japan) and expressed as a percentage of the aorta.

### Histology and immunohistochemistry

For the microscopic quantification of lesions, the aortic arch of each rabbit was cut into 8 to 10 sections (4 μm) as previously described [25]. To evaluate the microscopic lesion area of each aorta, all sections were stained with hematoxylin and eosin (HE), and measured by the image analysis system described above. For microscopic evaluation of cellular components in the lesions, serial paraffin sections of the aorta were immunohistochemically stained with the following antibodies (Abs) against macrophage (MФ) (RAM11, Dako, Carpinteria, CA, USA) and smooth muscle cells (SMC) (α-smooth muscle actin, Thermo Fisher Scientific Pierce, Rockford, IL, USA). Secondary Abs included anti-murine IgG (Beijing Zhongshan Biotechnology, Beijing, China) for MФ and SMC staining [25].

### Lesion type and quantitation

We analyzed whether the combined drug treatment had any effect on the progression of atherosclerosis according to American Heart Association guidelines in which atherosclerotic lesions are divided into I-VI morphologically characteristic types [26, 27]. To quantify lesion types, the total length of each lesion in the aortic arch was calculated in three groups using a method as reported in our previous study [28].

### Statistical analysis

The statistical analyses were carried out by one-way ANOVA followed by LSD test using the SPSS 13.0 software. In all cases, data were expressed as the mean ± SEM. *P* values less than 0.05 were considered statistically significant.

## Results

### Plasma lipid levels

**Fig. 1:**
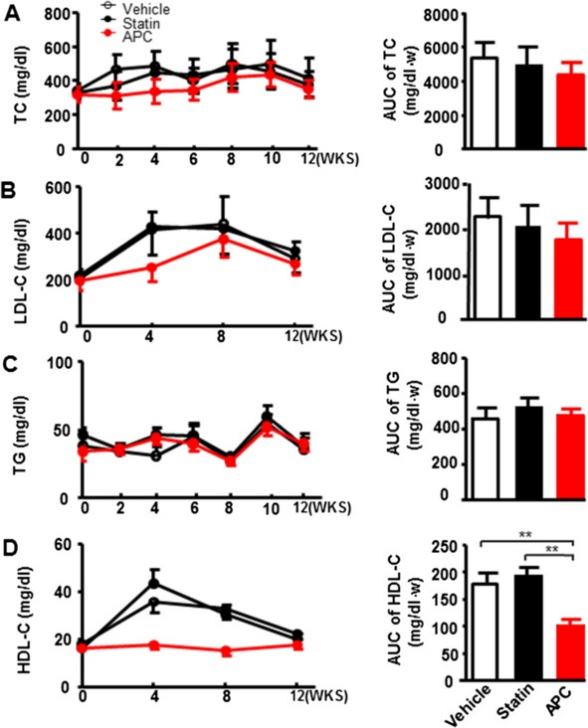
Plasma lipid levels. Plasma cholesterol total cholesterol (TC) (**a**), low-density lipoprotein cholesterol (LDL-C) (**b**), triglyceride (TG) (**c**), and high-density lipoprotein cholesterol (HDL-C) levels (**d**) were measured every 2 or 4 weeks. Data are expressed as the mean ± SEM, *n* = 10 for each group. **P < 0.01 vs. vehicle or statin treatment group

As shown in Fig. 1, the plasma levels of TC, LDL-C and TG were not significantly different among three groups, while the HDL-C level was significantly lower in APC group than vehicle and statin groups, respectively. Overall, in this study, statin and APC treatment did not affect plasma TC and TG levels.

The drug concentrations in each diet were confirmed by gas chromatography or high-performance liquid chromatographic as shown in Table 1. There was no difference in food intake or body weight among all groups (data not shown).

**Table 1:** Drug concentrations in diet from each group

Group	Atorvastatin	Probucol	Cilostazol
Vehicle	-	-	-
Atorvastatin	0.0037 %	-	-
APC	0.0039 %	0.2654 %	0.2491 %

### Gross lesion of aortic atherosclerosis

**Fig. 2:**
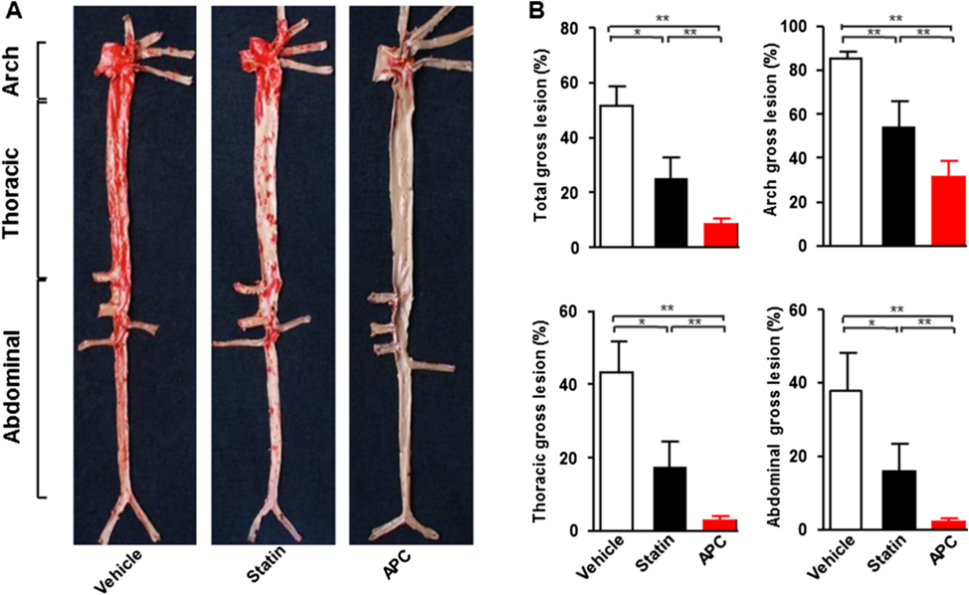
Aortic atherosclerotic lesions. Aortic trees were stained with Sudan IV (**a**) and the lesion area was calculated in different parts of aorta (**b**). Data are expressed as the mean ± SEM, *n* = 10 for each group. *P < 0.05, **P < 0.01 vs. vehicle or statin treatment group

In this experiment, even in the absence of a significant lipid-lowering effect, both statin and APC treatment led to a significant decrease in atherosclerotic gross lesions. Total atherosclerotic gross lesions were notably reduced by 85 % in APC treatment (*P* < 0.01) and 74 % in statin (*P* < 0.05) compared with vehicle (Fig. 2a and b). A similar reduction was found in all parts of the aortic tree, including the aortic arch, thoracic and abdominal aortas in APC compared to vehicle and statin groups (Fig. 2a and b). Apparently, the anti-atherogenic effect of combined triple drug therapy was efficacious than statin alone. Furthermore, this anti-atherogenic effect was not dependent of the lipid-lowering function of statins.

### Histological examination

**Fig. 3:**
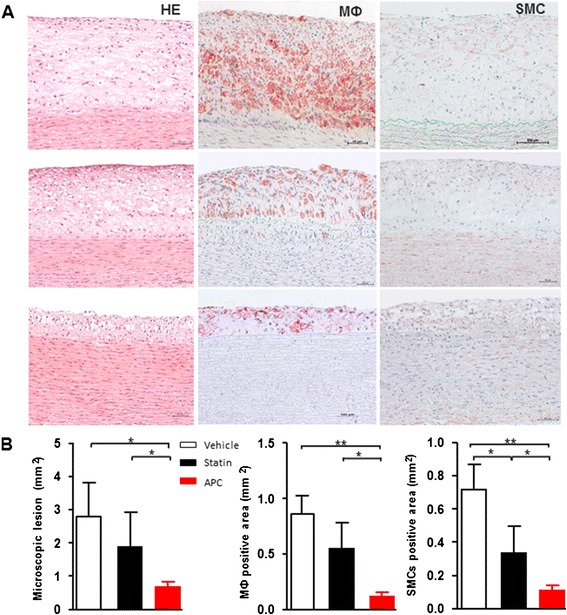
Histological analysis of aortic atherosclerosis. Representative micrographs of the intimal lesions and cellular components (**a**) and morphometric analysis of intimal lesions, lesion positive area of macrophage (MФ) and smooth muscle cells (SMCs) in these lesions (**b**). Data are expressed as the mean ± SEM, *n* = 10 for each group. *P < 0.05, **P < 0.01 vs. vehicle or statin treatment group

To quantify the size and components of atherosclerotic lesions, we measured the aortic arch sections using pathological analysis. Histological and immunohistochemical studies revealed that the intimal lesions and the positive area of MФ and SMC in statin group were reduced compared with the vehicle (Fig. 3). APC treatment significantly decreased the intimal lesions and the positive area of MФ and SMC compared with the vehicle (*P* < 0.05 or *P* < 0.01). Importantly, we found that the intimal lesions and the positive area of MФ and SMC in the APC group were further reduced compared with statin group (*P* < 0.05) (Fig. 3). These results suggest that APC treatment is more efficacious in inhibiting the intimal lesions and MФ and SMCs than stain treatment alone.

### Lesion type analysis

**Fig. 4:**
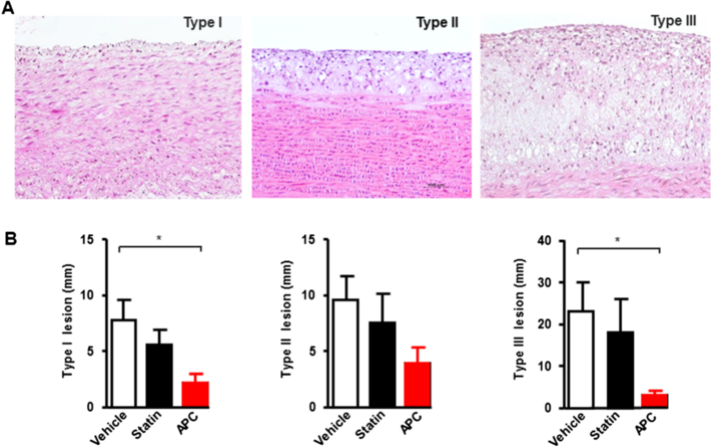
Lesions type analysis. Lesion types of the aortic arch were analyzed by microscope in each group (**a**). Lesion types I-III was quantified, respectively (**b**). Data are expressed as the mean ± SEM, *n* = 10 for each group. *P < 0.05 vs. vehicle

We further quantified the lesion types of the aortic arch in each group. We found that atherosclerotic lesions in cholesterol-fed rabbits were composed of type I, II and III lesions (Fig. 4). Although all types of lesions were reduced in both statin and APC groups, statistical significance was only found in APC group compared with the vehicle group (Fig. 4). Type III represents the stage that links type II to advanced lesions [26, 27].

### Inflammatory and oxidation markers

**Fig. 5:**
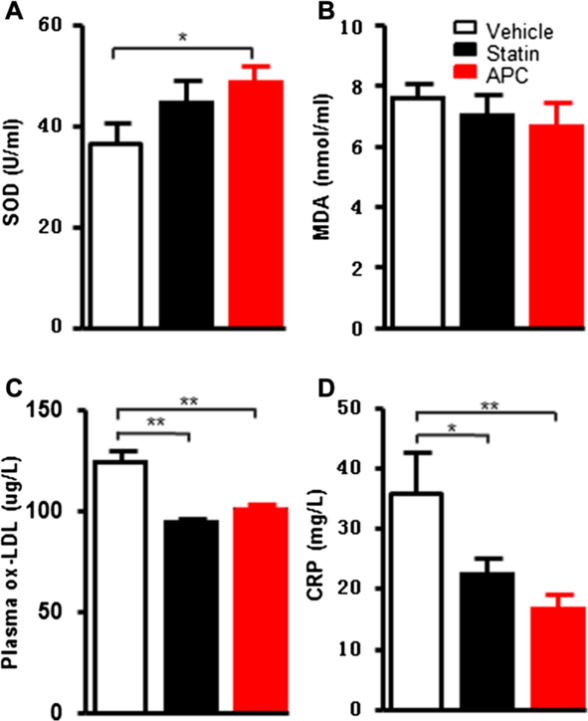
Oxidative stress and inflammation markers. Plasma superoxide dismutase (SOD) (**a**), malonaldehyde (MDA) (**b**), oxidized low-density lipoprotein (ox-LDL) (**c**) and C-reactive protein (CRP) levels (**d**) were measured at 12 weeks. Data are expressed as the mean ± SEM, *n* = 8 or 10 for each group. *P < 0.05, **P < 0.01 vs. vehicle or statin treatment group

To explore the possible mechanisms underlying APC anti-atherogenic effects, we measured plasma levels of SOD, MDA, ox-LDL and CRP at 12 weeks. As shown in Fig. 5, plasma SOD levels were significantly increased in the APC group (*P* = 0.04), while plasma MDA (even though not statistically different), ox-LDL and CRP levels were reduced in both APC and statin groups compared to vehicle (Fig. 5a-d).

## Discussion

Risk factors for atherosclerosis, such as dyslipidemia, inflammation, oxidative stress, abnormal levels of coagulant and central obesity often co-exist [29]. The present study provides evidence that the combined drug therapy consisting of probucol, cilostazol and atorvastatin markedly enhances the statin anti-atherogenic effect independent of a lipid-lowering manner in moderately hypercholesterolemic rabbit model.

In this experiment, rabbit model had moderate hypercholesterolemia (plasma TC = 350–500 mg/dl) compared to our previous studies (rabbit plasma TC = 800–1200 mg/dl) [19, 24, 25, 30, 31]. We used this model to investigate the effect of combined drug treatment in the early stages of atherosclerosis and found that, except for HDL-C, plasma lipids were not affected after 12 weeks of drug treatment. This was possibly due to the “relatively low” hypercholesterolemia baseline in these rabbits. However, APC treated group had lower HDL-C levels, which was caused by the presence of probucol. In spite of this, HDL functions were not impaired but were actually more efficient for reverse cholesterol transport(RCT) [32–35]. Previous studies have revealed that cholesterol ester transfer protein (CETP) plays a crucial role in mediating HDL functions and probucol may enhance reverse cholesterol transport by increasing CETP expression. HDLs mediated by enhanced CETP activity showed potentially anti-atherogenic functions [34]. In our previous study, we measured hepatic LDL receptor, SR-B1, ABCA1, CETP mRNA and protein expression levels and found that all drug treatment groups significantly increased hepatic LDL receptor mRNA expression by which hepatic uptake of LDLs would be enhanced (data not shown). Interestingly, we found combination treatment synergistically increased CETP mRNA and protein expression level in liver (data not shown). CETP as a plasma glycoprotein transfers CE from HDL to apoB-containing lipoproteins in exchange for triglycerides. Thus, directly through hepatic SR-B1 receptor uptake or indirectly through transfer of HDL-CE to ApoB-containing lipoproteins with subsequent receptor-mediated hepatic uptake, CETP could contribute significantly to the RCT pathway [36, 37].

Statins dramatically reduced cardiovascular events in patients with normal lipid levels or without established ASCVD, independently from lipid-lowering properties [38]. Moreover, plasma cholesterol lowering does not necessarily lead to protection against cardiovascular disease. In the present study, the attenuation of atherosclerotic lesions in APC treated group was independent of lipid-lowering function. These findings may be consistent with the “Mevalonate hypothesis” proposed recently [39] and further studies are needed for verifying. The anti-atherosclerosis in APC treatment may be due to their multiple pharmacological properties, such as enhanced anti-inflammatory and anti-oxidant effects in APC treatment group. Elevations in inflammatory markers, such as CRP, prospectively define the risk of atherosclerotic complications [31, 40, 41]. In the present study, plasma levels of CRP and ox-LDL were notably reduced in APC treated group compared to vehicle. Furthermore, SOD levels were significantly increased, while MDA levels simultaneously decreased in the APC treated group compared to vehicle. Statins as well as cilostazol, also known as platelet-activating factor inhibitors, play an important role in the crosstalk of dyslipidemia, inflammation and atherogenesis [42–46]. In the moderate hypercholesterolemia rabbit model, we found that combination treatment (statins, cilostazol and probucol) decreases atherogenesis via pleiotropic effects, such as anti-inflammation, anti-oxidation and inhibition of platelet-activating factor.

Although it remains to be verified clinically whether combined APC treatment exhibits a “potent anti-atherogenic function”, it seems that APC more strongly attenuates the progression of atherosclerosis than statin alone in cholesterol-fed rabbits. These insights may provide us with a new concept with which to effectively delay the occurrence of cardiovascular events by APC combined drug treatment in the early stages for those patients with or without established ASCVD.
